# Lesion mapping in acute stroke aphasia and its implications for recovery

**DOI:** 10.1016/j.neuropsychologia.2018.03.036

**Published:** 2018-07-01

**Authors:** Stephanie J. Forkel, Marco Catani

**Affiliations:** a Natbrainlab, King's College London, Department of Neuroimaging, Institute of Psychiatry, Psychology, and Neuroscience (IoPPN), De Crespigny Park, London SE5 8AF, United Kingdom; b Natbrainlab, Department of Forensic and Neurodevelopmental Sciences and Sackler Institute of Translational Neurodevelopment, Institute of Psychiatry, Psychology, and Neuroscience (IoPPN), King's College London De Crespigny Park, London SE5 8AF, United Kingdom

**Keywords:** Lesion mapping, VLSM, White matter atlas, Tractography, Acute stroke, Aphasia recovery

## Abstract

Patients with stroke offer a unique window into understanding human brain function. Mapping stroke lesions poses several challenges due to the complexity of the lesion anatomy and the mechanisms causing local and remote disruption on brain networks. In this prospective longitudinal study, we compare standard and advanced approaches to white matter lesion mapping applied to acute stroke patients with aphasia. Eighteen patients with acute left hemisphere stroke were recruited and scanned within two weeks from symptom onset. Aphasia assessment was performed at baseline and six-month follow-up. Structural and diffusion MRI contrasts indicated an area of maximum overlap in the anterior external/extreme capsule with diffusion images showing a larger overlap extending into posterior perisylvian regions. Anatomical predictors of recovery included damage to ipsilesional tracts (as shown by both structural and diffusion images) and contralesional tracts (as shown by diffusion images only). These findings indicate converging results from structural and diffusion lesion mapping methods but also clear differences between the two approaches in their ability to identify predictors of recovery outside the lesioned regions.

## Highlights

• Lesion mapping methods determine different lesion extents and associated outcomes.
• Voxel-based methods are sensitive to lesions associated with aphasia recovery.
• Diffusion data can reveal anatomical predictors of recovery beyond the lesion.

## 1 Introduction

Stroke imaging has greatly contributed to our current understanding of the anatomy of higher cognitive functions, including language. Most stroke studies are conducted in the chronic stage for practical reasons, but also to reduce the effect of clinical fluctuations, anatomical and metabolic changes, and possible functional reorganisation occurring between the acute and subacute phase (Baldo et al., 2016, Dronkers, 2000, Fridriksson et al., 2010, Hillis et al., 2000, Price, 2000, Price, 2012, Price and Friston, 2002, Turken and Dronkers, 2011b). In recent times, the increased availability of magnetic resonance imaging (MRI) scanners with clinically feasible acquisition sequences has led to an increasing number of studies focusing on acute stroke (Forkel et al., 2014, Hillis and Heidler, 2002, Hillis et al., 2001, Saur et al., 2006). Compared to chronic stroke, acute stroke imaging offers several advantages (Karnath and Rorden, 2012). For example, inter-individual differences in structural anatomy and cognitive functioning can be best studied in the acute stage, as the lesion has not yet caused full degeneration of anatomical structures and patients have yet to develop their own cognitive strategies to overcome their deficits. Additionally, some conditions such as anosognosia for hemiplegia and visuospatial neglect are more prominent in the acute stages and their clinical progression can be better studied in early post-stroke days (Besharati et al., 2014, Besharati, 2016, Cutting, 1978, Orfei et al., 2009). Perhaps the most important advantage of acute stroke imaging lies in the possibility of identifying early anatomical predictors of recovery, especially in longitudinal studies (Forkel et al., 2014).

**Fig. 1:**
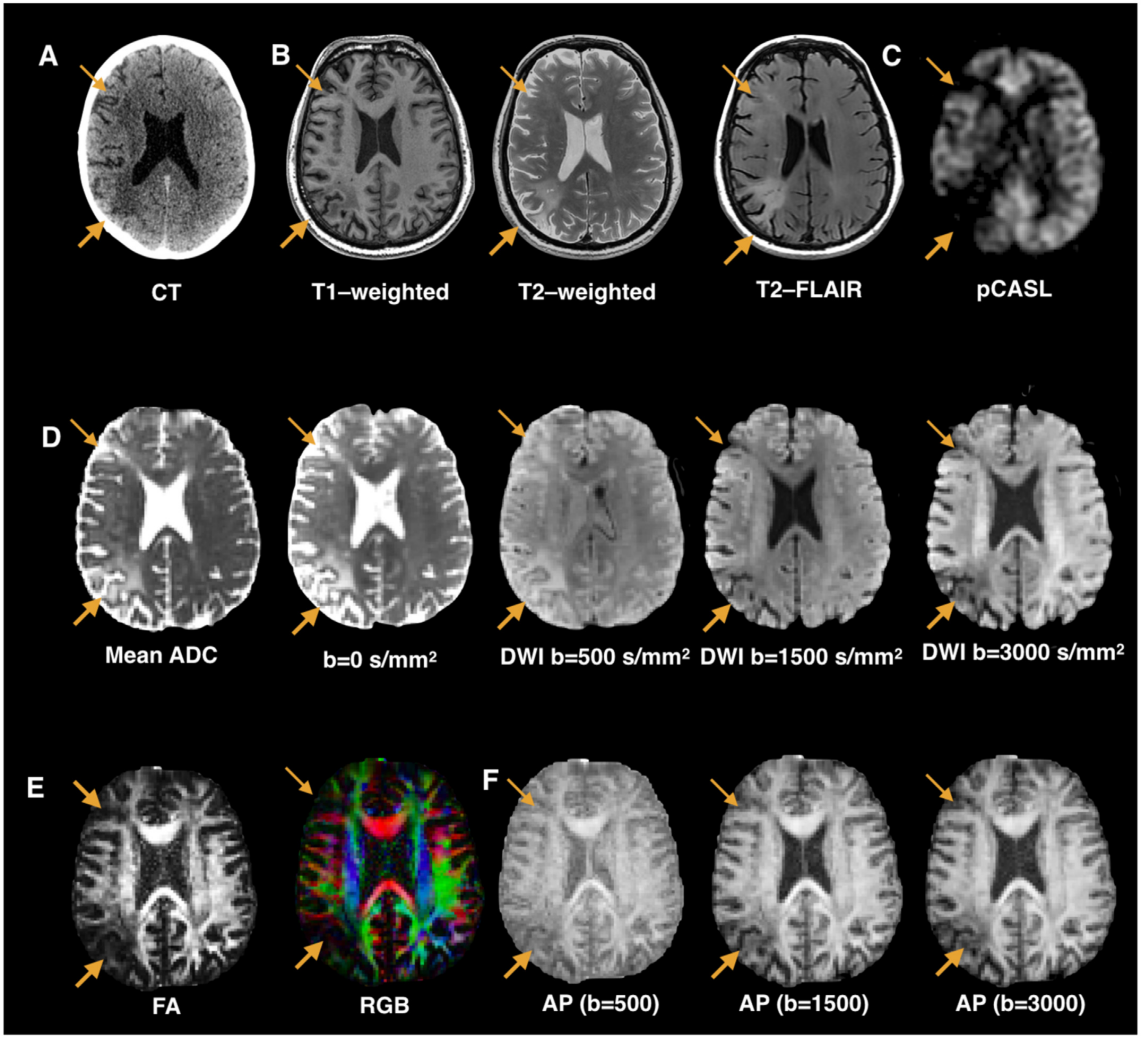
Structural, perfusion, and diffusion images acquired in a single patient with acute stroke and aphasia. The arrows indicate a large ischemic lesion in the left parieto-occipital region and smaller ischemic lesion in the frontal lobe. A. Non-contrast computerised tomography (CT) scan on admission that shows altered signal only in posterior lesion. B. Conventional structural MRI sequences where - similarly to the CT scan - the frontal lesion is barely visible on these contrasts. C. Pulsed continuous arterial spin labelling (pCASL) shows reduced cortical and subcortical blood perfusion in both posterior and anterior regions of the left hemisphere. D. Mean apparent diffusion coefficient (ADC) map and multishell (b-value = 0/500/1500/3000 s/mm2) diffusion weighted imaging (DWI) maps. The boundaries of the lesions vary according to the value of the diffusion weighting. A lower b-value (i.e. less diffusion-weighting) is more sensitive to fast diffusion and therefore to very early ischemic changes, for example due to oedema, but it is also affected by partial volume effects from cerebrospinal fluid. Higher b-value images are more sensitive to slow diffusion and better tissue contrast, for example, between grey and white matter or different white matter tracts. E. Diffusion tensor imaging permits for the generation of maps with additional anatomical information not available from structural MRI images. The fractional anisotropy (FA) map encodes scalar values between zero (indicating diffusion isotropy) and 1 (high degree of diffusion anisotropy). Low FA is typical of voxels containing fluid, cortex, or crossing fibres. The red-green-blue (i.e. RGB) colour-coded map indicates the main orientation of the tensor and therefore the average orientation of the fibres contained in each voxel. By convention red identifies a medial-to-lateral orientation (i.e. commissural pathways), green a longitudinal orientation (i.e. long association pathways), and blue a vertical orientation (i.e. projection pathways). F. Anisotropic power (AP) maps quantify the absolute amount of anisotropic information present in any measured High Angular Resolution Imaging (HARDI) signal profile and is more robust to noise compared to other anisotropy maps. With a higher b-value the anatomical detail encoded in the AP maps increases with better differentiation between lesioned and healthy tissue.

The most widely used imaging modality in stroke is computerised tomography (CT) owing to its ability to quickly determine the presence of haemorrhage or ischemia and inform individualised treatment pathways (Fig. 1A). While this makes CT a very feasible imaging technique in the clinical routine, its low spatial resolution and reduced sensitivity to early ischemic changes has progressively favoured the use of MRI for research studies (Wall et al., 1982). Structural (T1-weighted, T2-weighted, fluid attenuated inverse recovery; FLAIR, etc.), perfusion, and diffusion imaging are the most common MRI modalities currently applied in stroke research and to some extent in clinical practice. Structural MRI sequences provide high anatomical resolution, which helps to optimise lesion identification and delineation (Fig. 1B) (Bates et al., 2003, Dronkers et al., 2004, Fiez et al., 2000). In contrast, perfusion MRI provides low spatial but high temporal resolution images, which makes it highly sensitive to early blood flow changes (Fig. 1C) (Hillis et al., 2001, Hillis and Heidler, 2002).

Similarly to perfusion, diffusion MRI has a low spatial resolution compared to structural sequences but good temporal resolution, which improves the sensitivity to acute lesion detection to over 95% (Birenbaum et al., 2011). Diffusion can, therefore, visualise early ischemic changes that may not otherwise be detectable with CT or more conventional structural MRI and shows a high accuracy in predicting final infarct size (Brant-Zawadzki et al., 1985, Schellinger et al., 2010). However, spatial and temporal resolutions of different diffusion modalities vary widely according to the choice of acquisition parameters (Fig. 1D). Mean apparent diffusion coefficient (ADC) images are the most widely used in clinical routine for their high sensitivity to early ischemia and fast acquisition time (i.e. only three diffusion-encoding directions are necessary), yet their spatial resolution is rather low (Le Bihan et al., 1986, Warach et al., 1995) (Fig. 1D). Acquiring diffusion images along at least six directions permits the calculation of the diffusion tensor and the generation of fractional anisotropy (FA), mean diffusivity (MD) and colour-coded (i.e. RGB maps) maps (Pajevic and Pierpaoli, 1999). Similar to ADC, all diffusion tensor-based maps are very sensitive to the early ischemic changes and reorganisation occurring during subacute and chronic stages (Moseley et al., 1990). FA and RGB maps offer additional information on white matter organisation, such as degree of cohesiveness of fibres (i.e. FA maps) or fibre orientation (i.e. RGB maps). Compared to structural MRI images, diffusion maps based on the tensor model have low spatial resolution, which makes them unsuitable for lesion delineation. FA values are also significantly reduced in regions containing fibre crossing, in which it is difficult to distinguish between normal and pathological tissue. To overcome the problems of tensor-based diffusion maps, anisotropic power (AP) maps have been recently proposed (Dell'Acqua et al., 2014, Fig. 1F). These maps are based on High Angular Resolution Diffusion Imaging (HARDI) methods that can be applied to diffusion data acquired at higher b-values (e.g. > 1500) and with a higher number of directions (Dell'Acqua et al., 2010, Dell’Acqua 2012). The AP maps offer the advantage of combining the high sensitivity to early ischemia typical of the diffusion sequences with an increased signal in regions with fibre crossing and a spatial resolution similar to clinical T1-weighted images. Finally, all tensor and HARDI methods can be used to generate three-dimensional representations of white matter pathways using tractography algorithms (for a general review see Dell’Acqua and Catani, 2012), which allows for the study of remote lesion effects on the connectional anatomy within the ipsilesional and contralesional hemispheres (Catani and ffytche, 2005, Catani et al., 2005).

The availability of the above imaging modalities has widened our ability to map the complexity of stroke lesions and extend the clinical-anatomical correlation to both the cortex and white matter pathways (Catani et al., 2012). The importance of including white matter, especially in the analysis of stroke recovery studies, has been recently highlighted by several groups (Bartolomeo and Thiebaut de Schotten, 2016, Corbetta et al., 2015, Forkel et al., 2014, Yourganov et al., 2016; Marewba et al., 2017; Foulon et al., 2018). Structural MRI is traditionally used for lesion delineation and provides an estimate of the lesion location, size, and extent to both cortical and subcortical regions. However, it is often difficult to use structural MRI to identify damage to individual pathways and quantify their degree of disconnection. This information can be indirectly derived by combining lesion delineation based on structural MRI with white matter atlases derived from either histological studies or diffusion imaging (Eikhoff et al., 2007; Catani and Thiebaut de Schotten, 2008; Hua et al., 2008; Mori et al., 2005; Rojkova et al., 2015; Wakana et al., 2004; Thiebaut de Schotten et al., 2011). However, a direct estimate of white matter changes and damage to specific tracts can only be obtained using diffusion imaging tractography.

Two main approaches to mapping white matter damage with diffusion data are available. Similar to structural MRI, diffusion images can be analysed using a voxel-based approach. A method specifically developed for group-level analysis of diffusion data is tract-based spatial statistics (TBSS, Smith et al., 2004, Smith et al., 2007), which despite the name, is a voxel-based approach with most of the limitations that affect all voxel-based methods (Jones and Cercignani, 2010). The other approach to diffusion data is tractography, which permits to reconstruct the trajectory of individual white matter pathways in single patients (Catani et al., 2002; Catani and Thiebaut, 2008).

Two possible mechanisms have been suggested to explain the contribution of white matter pathways to recovery. First, the number of tracts affected by the stroke is highly correlated with the severity of deficits in multiple behavioural domains and functional recovery (Corbetta et al., 2015, Umarova et al., 2017, Yourganov et al., 2016; Marewba et al., 2017, Thiebaut de Schotten and Fouloun, 2018). Second, the individual anatomy of white matter pathways in the contralesional hemisphere is an important predictor for aphasia recovery, suggesting the possibility of some functions being partially taken over or compensated by a contralateral language network (Forkel et al., 2014). Due to the high anatomical variability of the contralateral language pathways in the general population (Catani et al., 2007), these recovery mechanisms may occur only in a subgroup of patients and may contribute to explain the high heterogeneity in stroke recovery in patients with similar demographics and lesion characteristics (Forkel et al., 2014)

In this study, we perform lesion-symptom correlations using structural and diffusion maps and employ standard voxel-based approaches based on lesion overlay (i.e. voxel-based lesion symptom mapping, VLSM) and diffusion specific methods such as TBSS and tractography. Our aim is to provide preliminary evidence of the advantages and limitations of a quantitative approach to mapping white matter damage in acute stroke and its impact on language recovery.

## 2 Methods

### 2.1 Patients

18 aphasic stroke patients with a first-ever left hemisphere lesion (mean age: 63.39 ± 18.44 years, range 28 – 89 years, 6 females) were consecutively recruited from the hyperacute stroke unit at King's College Hospital in London between 2009 and 2012. The detailed demographics of this cohort are reported in Table 1.

**Table 1:** Patient demographics and clinical presentation.

				**Aphasia type**^b^		**Aphasia severity (AQ)**	
**Sex**	**Age**^a^	**Ethnicity**	**Stroke type**	**Baseline**	**Follow-up**	**Baseline**	**Follow-up**
F	87	White British	Infarction	Anomic	Recovered	75.90	95.20
M	28	White Irish	Infarction	Transcortical motor	Recovered	45.00	96.20
M	72	White Irish	Infarction	Transcortical motor	Anomic	67.00	81.40
M	70	White British	Infarction	Broca	Anomic	42.00	91.90
F	69	White British	Infarction	Global	Anomic	11.50	73.30
F	81	White British	Infarction	Anomic	Anomic	79.50	87.90
M	75	White British	Haemorrhage	Wernicke	Wernicke	15.40	73.50
F	44	White British	Infarction	Broca	Anomic	58.40	87.20
M	59	Black Caribbean	Infarction	Broca	Anomic	32.80	81.00
M	50	White British	Infarction	Global	Transcortical motor	4.70	83.10
F	71	Black Caribbean	Infarction	Global	Anomic	21.6	69.70
M	44	White British	Infarction	Anomic	Recovered	79.16	95.60
M	86	British White	Infarction	Conduction	n/a	60.83	n/a
M	49	White British	Infarction	Broca	Anomic	19.20	89.20
M	89	British White	Infarction	Global	n/a	17.60	n/a
M	79	White British	Infarction	Broca	Anomic	59.00	81.10
F	44	White British	Infarction	Broca	Anomic	6.03	92.20
M	44	Indian British	Infarction	Anomic	Anomic	78.30	92.30

F: female, M: male, AQ: Aphasia Quotient.

a Age at onset shown in years.

b Aphasia type classification based on WAB-R.

Patients were assessed on admission using the revised Western Aphasia Battery (WAB-R; Kertesz, 2007), which provides an estimate of aphasia severity (aphasia quotient, AQ). 16 patients were available for follow-up assessments six months after symptom onset. Inclusion criteria were: (i) right-handedness based on Edinburgh Handedness Inventory (Oldfield, 1971); (ii) first ever left middle cerebral artery stroke with no previous presence of brain infarction; (iii) presence of aphasia as confirmed by the WAB-R bedside screening; (iv) no previous neurological or psychiatric diagnoses; (v) medically stable to tolerate ambulance transport; (vi) absence of MRI contraindications; and (vii) English native speaker. All patients or their next of kin gave written informed consent. The study was approved by the Wandsworth Ethical Research Committee (09/H0803/95) and the local review board (KCH1700).

### 2.2 Neuroimaging acquisition and processing

Baseline MRI data was acquired within two weeks from symptom onset using a 3 T HDx GE scanner (General Electric) equipped with an 8-channel radio frequency receiver head coil. For each subject, a high-resolution structural T1-weighted volume (1*1*1 mm) of the whole brain was acquired. Diffusion imaging data were acquired using a spin echo, single shot EPI pulse sequence optimised for subjects with high risk of movement during the scan (Forkel et al., 2014). This sequence consisted of two consecutive scans of 30 diffusion-weighted directions (b-value 1500 mm2/s) combined for a total of 60 directions and seven non-diffusion weighted volumes. Matrix size was 128*128*60 and voxel size was 2.4*2.4*2.4 mm. Peripheral gating was applied to avoid brain pulsation artefacts.

Diffusion imaging data were pre-processed using ExploreDTI (www.exploredti.org) and corrected for eddy current and motion artefacts through iterative correction to the seven interleaved non-diffusion weighted volumes. For each subject, data quality was visually inspected, and no participant had to be excluded. The data was processed in Startrack (https://www.mr-startrack.com), which also generates diffusion-based maps, including FA and AP maps (Dell'Acqua et al., 2014). Whole brain tractography was performed from all brain voxels with FA > 0.2. Streamlines were propagated with a stepsize of 1 mm, using Euler integration and b-spline interpolation of the diffusion tensor field (Basser et al., 2000). Where FA was < 0.2 or the angle between two consecutive voxels exceeded 45 degrees, streamline propagation was stopped.

### 2.3 Neuroimaging analysis

#### 2.3.1 Voxel-based percentage lesion overlay mapping

Lesions were manually (SF) delineated on both native-space T1-weighted images and AP maps for each patient and were subsequently converted to a common space (Brett et al., 2001, Mah et al., 2014, Rorden et al., 2012). A lesion overlay percentage heat map was calculated from all lesions and superimposed on a template brain using MRICron (Rorden et al., 2007a, Rorden et al., 2007b). These maps highlight damaged areas with the highest degree of spatial overlap in a group of patients. For T1-based lesion analysis one patient had to be excluded as no T1-weighted scan was acquired.

#### 2.3.2 Atlas-based mapping of white matter disconnection

For each lesion mask defined on T1-weighted or AP maps, the intersection between an individual lesion mask and a percentage tract map from an atlas of known white matter tracts (Rojkova et al., 2015) was defined using Tractotron as implemented within the BCBtoolkit (http://www.toolkit.bcblab.com). Tractotron provides the probability of a lesioned voxel intersecting a specific tract of interest within a range between 0% (no streamlines intersect at that particular voxel for the tract of interest extracted from all subjects constituting the group of reference) and 100% (all healthy subjects have streamlines intersecting at that particular voxel for the tract of interest). Hence, the percentage provided by Tractotron does not reflect the probability of a disconnection but the probability of a lesion voxel overlapping with a voxel of high probability to contain a specific white matter pathway. This value is provided independently of the number of lesion voxels overlapping with the mask, which means that even a single lesion voxel intersecting a voxel with a 50% pathway overlap is sufficient for a pathway to be classified as having a 50% probability of being affected (Foulon et al., 2018). The data were taken to IBM SPSS, Version 24.0 for central tendency analysis at the group level with the behavioural data.

#### 2.3.3 Voxel-based lesion-symptom mapping (VLSM)

Binarised and normalised lesion masks were entered into a VLSM pipeline using the NPM program implemented in MRIcron (non-parametric mapping; http://www.cabiatl.com/mricro/npm/). VLSM analysis (including all patients, irrespective of aphasic classification) was run for the dependent continuous variable of 6-months aphasia severity (follow-up AQ) and controlled for overall lesion size. A non-parametric rank-order Brunner-Menzel analysis with voxel-based permutation (1000) was conducted (Rorden et al., 2007a, Rorden et al., 2007b). Only voxels where at least 10% of patients had damage were included in the analysis to avoid lowering statistical power by including infrequently damaged voxels whilst increasing the number of computed comparisons. Group-level results were then projected onto a high-resolution template in standard space.

#### 2.3.4 Tract-Based Spatial Statistics (TBSS)

Tract-Based Spatial Statistics, is a group level automatic analysis that employs a non-linear registration and generates a whole brain white matter skeleton based on the FA maps obtained from diffusion data (Smith et al., 2004, Smith et al., 2007). TBSS relies on a group mean FA map that is skeletonised to represent the core of the brain's white matter, which is common to all subjects within the studied cohort. Individual FA maps of our patients were aligned to a common target using non-linear registration and an average FA map was generated. The mean FA map was skeletonised through “thinning” and individual normalised FA maps were projected onto the skeleton to account for residual misalignment from the first step. Subsequent voxel-wise statistics are applied across subjects on the skeleton-space FA data. The data was statistically analysed in FSL (version 4) using Randomise (Winkler et al., 2014). This permutation method is used for thresholding statistical maps where the null distribution is unknown (i.e. non-parametric testing). This modelling and interference can be achieved by using a standard general linear model design setup (Nichols and Holmes, 2002). TBSS analysis was performed using follow-up AQ as the main neuropsychological variable of interest. Patient demographics (e.g. age, sex, lesion size) were treated as covariates and were defined as explanatory variables in the model. Values were entered ‘demeaned’ into the analysis; this means the value of interest is subtracted from the group mean.

#### 2.3.5 White matter tractography

Virtual dissections of the language pathways in both hemispheres were performed in TrackVis (www.trackvis.org). Regions of interest for both hemispheres were defined on FA maps in the patients’ native space using previously published criteria (Catani et al., 2005, Catani et al., 2007, Forkel et al., 2014, D'Anna et al., 2016). For the three segments of the arcuate fasciculus we used three regions of interest (ROI) delineated in the frontal, parietal and temporal perisylvian white matter (Barroso-Lopez et al., 2013; Forkel et al., 2014; Catani et al., 2016). Three regions of interest were also used to dissect the inferior fronto-occipital fasciculus (IFOF), the unicinate fasciculus (UF) and the inferior longitudinal fasciculus (ILF) (Catani and Mesulam, 2008; Forkel et al., 2014; D'Anna et al., 2016). The first ROI was delineated in the occipital lobe on a coronal plane just behind the parieto-occipital sulcus and the temporo-occipital notch, the second ROI was drawn on the white matter of the external/extreme capsule, and the third ROI in the white matter of the anterior temporal lobe. Streamlines between occipital ROI and the external/extreme capsule ROI were labelled as IFOF, streamlines between occipital and anterior temporal were identified as ILF, and streamlines between anterior temporal and external/extreme capsule ROI were labelled as UF. The frontal aslant tract (FAT) was defined by placing one region of interest in posterior white matter of the superior frontal gyrus and a second ROI in the posterior inferior frontal gyrus and ventral precentral gyrus (Catani et al., 2012a, Catani et al., 2012b; Thiebaut de Schotten et al., 2011; Catani et al., 2013a, Catani et al., 2013b).

The data were taken to IBM SPSS, Version 24.0 for statistical analysis. To control for the possibility that hemisphere size might be driving the volume of the white matter pathways, the tract volume was normalised by the hemisphere volume (normalised pathway volume = segment volume/hemisphere volume). The hemispheric volume was obtained using FMRIB Software Library package (FSL, http://www.fmrib.ox.ac.uk/fsl/). The normalised segment volume was then used for further analysis. The primary analysis employed a hierarchical linear regression. In this analysis, two models were defined with the longitudinal aphasia severity (AQ) at 6 months as the dependent variable. The first-level model included the variables identified by a backward elimination, namely age, gender and lesion size. In the second-level of the model the normalised volumes of the three segments of the arcuate fasciculus, the FAT, IFOF and uncinate fasciculus were separately added to the model.

## 3 Results

Patient details and demographics are available in Table 1.

Percentage lesion overlay maps, atlas-based mapping and VLSM analyses were performed for both T1-weighted and AP maps. TBSS and tractography were performed using FA maps.

### 3.1 Lesion mapping using T1-weighted MRI

**Fig. 2:**
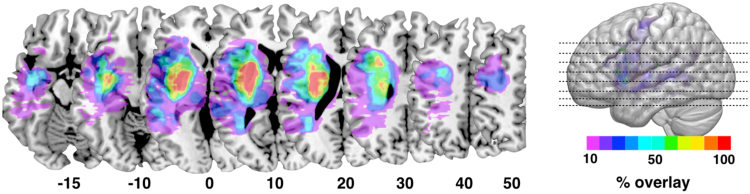
Percentage lesion overlay maps based on lesion masks delineated on T1-weighted images.

Fig. 2 shows the *percentage lesion overlay maps* based on individual acute T1-weighted scans. The area of maximal overlay across all patients is centred around the lenticular nucleus/external capsule and includes white matter voxels extending into the extreme capsule, internal capsule and the perisylvian white matter and cortex. These regions contain several white matter tracts that are difficult to distinguish based solely on visual inspection of the percentage lesion overlay maps.

An *atlas-based approach* was, therefore, applied to the lesion masks defined on T1-weighted images of each subject using Tractotron. The lesion mask overlapped with regions occupied by the inferior fronto-occipital fasciculus (IFOF; 12 patients) and uncinate fasciculus (9 patients) in the external/extreme capsule and the cortico-spinal tract (15 patients), the fronto-striatal (16 patients) and anterior thalamic projections (17 patients) in the internal capsule. In the perisylvian white matter all three segments of the arcuate fasciculus were affected, although to a different degree: long segment (12 patients), anterior segment (7 patients), and posterior segment (7 patients). Dorsally to the arcuate fasciculus, the second branch of the superior longitudinal fasciculus (SLF II; 13 patients) and the first branch (SLF I; 8 patients) were affected. Within the frontal lobe, the frontal aslant tract (FAT) was damaged in most patients (14 patients), whereas the frontal inferior longitudinal fasciculus (FIL; 9 patients), the frontal superior longitudinal fasciculus (FSL; 7 patients), and the fronto-orbitopolar tract (FOP; 2 patients) were affected in a smaller number of patients. Other damaged tracts included the anterior fronto-insular tracts, the cingulum (10 patients), the fornix (8 patients), and the inferior longitudinal fasciculus (ILF, 8 patients). The damaged commissural pathways included the corpus callosum (17 patients) and the anterior commissure (7 patients).

### 3.2 Lesion mapping based on diffusion data

**Fig. 3:**
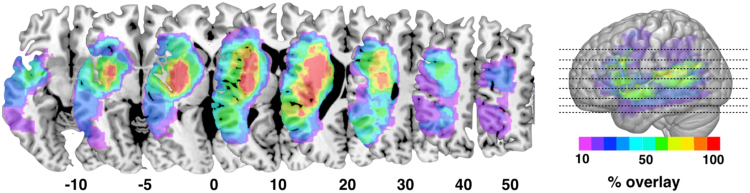
Percentage lesion overlay map based on lesion masks defined on anisotropic power (AP) images.

Similar to T1-weighted images, diffusion data can be analysed using voxel-based approaches. Fig. 3 shows the *percentage lesion overlay maps* based on the lesions defined on AP maps. Although the area of maximal overlay between T1-weighted and AP is very similar, the AP maps identified a larger area of overlay that include the head of the caudate nucleus, more extensive involvement of basal ganglia, and voxels in the white and grey matter of the anterior frontal, parietal, and temporal lobes.

The *atlas-based approach* applied to the AP lesion masks showed similar results to the T1-weighted images, although the extent of the overlap between lesions and tract masks and the number of patients with lesion in a particular tract was greater for the AP maps. This difference was particularly evident for the three segments of the arcuate fasciculus.

Diffusion data were also analysed using *tractography* reconstructions of the main language pathways. For each tract, the volume and FA were extracted and comparison between the two hemispheres carried out to identify affected tracts (Table 2). Statistically significant differences were observed for the volume of the anterior segment (p < 0.001) of the arcuate fasciculus and the inferior fronto-occipital fasciculus (p < 0.01). For the FA, statistically significant differences were observed for all tracts except the frontal aslant tract.

**Table 2:** Tractography analysis of the volume and fractional anisotropy (FA) of the language pathways in the left and right hemisphere.

	**Left hemisphere**	**Right hemisphere**	**Test statistic**
***Tract volume (mean/SD)***			
*Arcuate fasciculus, anterior segment*	8.32 ± 4.57	13.72 ± 4.47	t(16)−3.96, p = 0.001
*Arcuate fasciculus, posterior segment*	10.74 ± 3.10	8.43 ± 2.53	t(16) = 2.52, p = 0.02
*Arcuate fasciculus, long segment*	10.72 ± 5.44	7.79 ± 4.51	t(16) = 2.20, p = 0.43
*Frontal aslant tract*	6.57 ± 3.95	7.14 ± 2.49	t(16) = −0.62, p = 0.54
*Inferior fronto-occipital fasciculus*	13.02 ± 5.02	18.74 ± 3.88	t(16) = −4.09, p = 0.001
*Uncinate fasciculus*	7.72 ± 2.91	9.34 ± 2.61	t(16) = −1.91, p = 0.075
***Fractional anisotropy (mean/SD)***			
*Arcuate fasciculus, anterior segment*	0.39 ± 0.39	0.46 ± 0.44	t(15) = −5.163, p < 0.001
*Arcuate fasciculus, posterior segment*	0.40 ± 0.03	0.44 ± 0.03	t(16) = −3.89, p = 0.001
*Arcuate fasciculus, long segment*	0.43 ± 0.40	0.48 ± 0.23	t(15) = −5.85, p < 0.001
*Frontal aslant tract*	0.41 ± 0.43	0.43 ± 0.33	t(16) = −2.43, p = 0.027
*Inferior fronto-occipital fasciculus*	0.46 ± 0.03	0.49 ± 0.03	t(16) = −3.63, p = 0.002
*Uncinate fasciculus*	0.40 ± 0.04	0.45 ± 0.03	t(16) = −5.15, p < 0.001

### 3.3 Predicting recovery using T1-weighted MRI

**Fig. 4:**
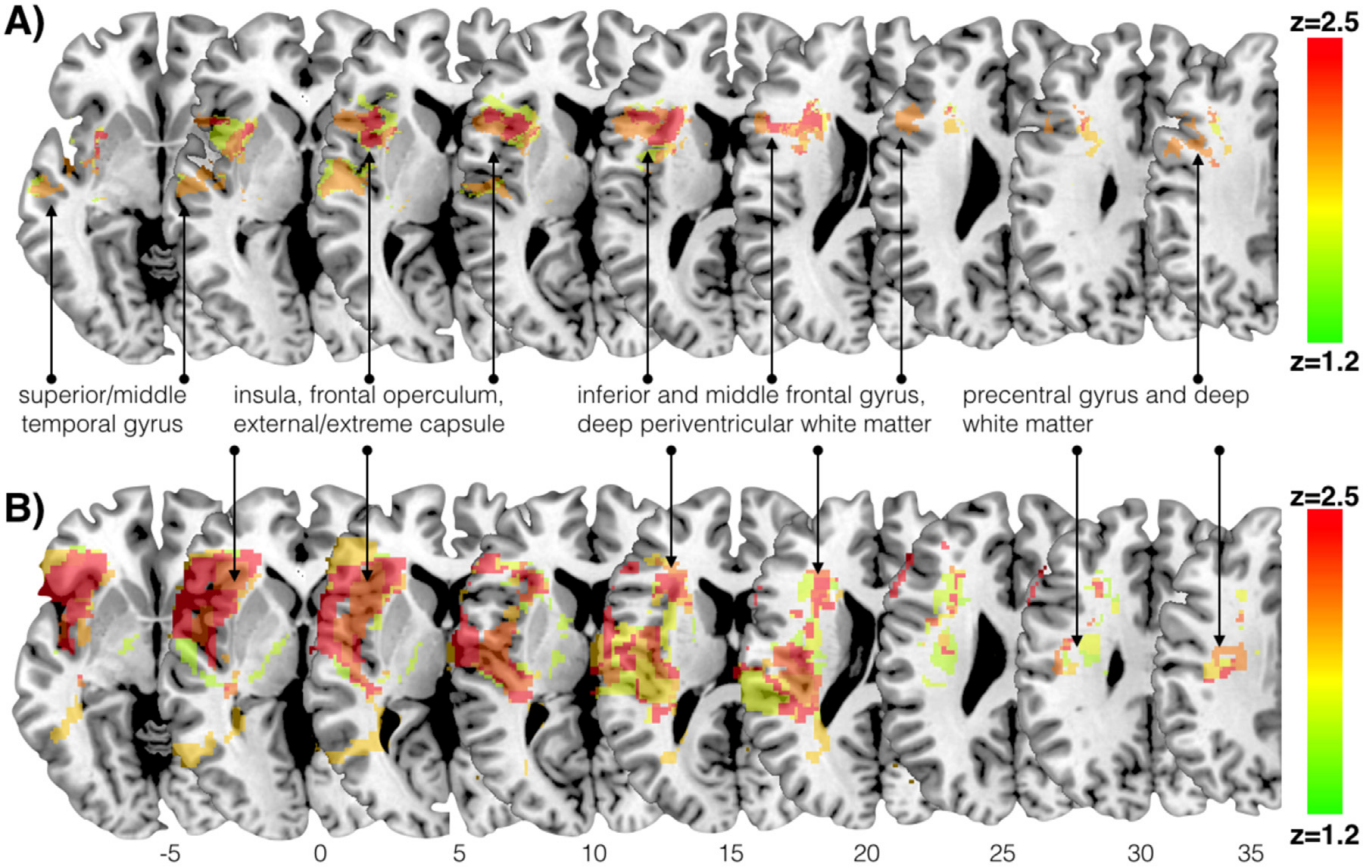
Voxel-based lesion symptom mapping (VLSM) analysis based on A) acute T1-wighted masks and B) AP masks indicating damaged voxels predicting aphasia severity at six months (longitudinal AQ). Results are not corrected for multiple comparisons (P < 0.05 for Z > 1.64). Axial slices are numbered according to MNI z coordinate. The AP-based analysis shows a larger territory of symptom-lesion association extending into posterior regions of the temporal and fronto-parietal lobes.

VLSM analysis using continuous scores for longitudinal aphasia severity at six months showed that lesions involving white matter voxels within the posterior and ventral region of the left frontal lobe were significantly associated with severity of language deficits at follow-up (Fig. 4A). Other significant white matter clusters were located in the posterior region of the superior temporal gyrus and Heschl's gyrus. The cluster with the maximum (Z = 2.5, p ≤ 0.05) matched the region around the anterior insula and frontal operculum that corresponds in part to the area of maximal overlay indicated on the T1-weighted percentage lesion overlay maps. Lesions to the lenticular nucleus which showed maximal overlay on the T1-weighted percentage lesion overlay maps were not associated with aphasia severity at six months.

The *atlas-based approach* was used with the T1-based lesion masks to compare longitudinal aphasia severity between patients with high and low likelihood of tract disconnection. Owing to a small patient cohort we did not opt for the approach used in the literature whereby patients are divided according to a threshold of 50% overlap (i.e. patients whose lesion mask intersected at least one voxel of the atlas maps with >50% overlap, are deemed to have a probability of disconnection of the tract of interest above chance) (Thiebaut de Schotten et al., 2014); Foloun et al., 2018. Instead, we used a median split to compare patients with high and low likelihood of tract disconnection. For statistical analysis, only white matter pathways which have been previously implicated in aphasia were considered. A Mann-Whitney-*U* test indicated that damage to the anterior segment of the arcuate fasciculus and the frontal aslant tract was associated with more severe aphasia symptoms at baseline (Table 3). The association between symptoms at baseline and damage to the long segment of the arcuate fasciculus did not survive correction for multiple comparisons. The same analysis was repeated for the longitudinal aphasia severity and none of the tracts were significantly associated with aphasia severity.

**Table 3:** Comparison of baseline and longitudinal aphasia severity between patients with high and low likelihood of disconnection for a specific tract based on atlas-based approach of T1-weighted lesion masks.

	**Above median (rank, n)**	**Below median (rank, n)**	**Test statistic**
***Arcuate fasciculus, long segment***			
*Aphasia severity (AQ), baseline*	11.62 (8)	6.67 (9)	U = 15, p = 0.046
*Aphasia severity (AQ), 6 months*	10.43 (7)	5.88 (8)	U = 11, p = 0.54
***Arcuate fasciculus, anterior segment***			
*Aphasia severity (AQ), baseline*	13.5 (8)	5 (9)	U = 0, p < 0.001
*Aphasia severity (AQ), 6 months*	10 (7)	6.25 (8)	U = 14, p = 0.121
***Arcuate fasciculus, posterior segment***			
*Aphasia severity (AQ), baseline*	8.12 (8)	9.78 (9)	U = 43, p = 0.541
*Aphasia severity (AQ), 6 months*	6.5 (8)	9.71 (7)	U = 40, p = 0.189
***Frontal aslant tract***			
*Aphasia severity (AQ), baseline*	13.33 (6)	6.64 (11)	U = 39, p = 0.007
*Aphasia severity (AQ), 6 months*	11.20 (5)	6.4 (10)	U = 9, p = 0.055
***Inferior fronto-occipital fasciculus***			
*Aphasia severity (AQ), baseline*	8.5 (6)	9.27 (11)	U = 36, p = 0.808
*Aphasia severity (AQ), 6 months*	9.4 (5)	7.3 (10)	U = 18, p = 0.440
***Uncinate fasciculus***			
*Aphasia severity (AQ), baseline*	9.89 (9)	8 (8)	U = 28, p = 0.481
*Aphasia severity (AQ), 6 months*	9.57 (7)	6.62 (8)	U = 17, p = 0.232

In addition, we also studied the collinearity of our measures and observed a statistically significant correlation between baseline severity and likelihood of tract damage for the anterior (r = –0.76, p < 0.001) and long segment (r = –0.66, p = 0.004) of the arcuate fasciculus, and the frontal aslant tract (r = –0.73, p < 0.001). No statistically significant correlations were observed with the severity at follow-up.

### 3.4 Predicting recovery using diffusion data

*VLSM* analysis of longitudinal aphasia severity was repeated using lesion masks drawn on AP maps. Whilst the maximal overlay (z = 3.06, p ≤ 0.05) was in the anterior insula and frontal opercular region, similar to the VLSM analysis of T1-weighted images, a larger number of voxels in the posterior temporal region, external capsule, and posterior frontal lobe were associated with longitudinal deficits and the AP maps (Fig. 4B).

The *atlas-based approach* for the AP-based lesion masks identified the anterior segment of the arcuate fasciculus and the frontal aslant tract as statistically significant associated with baseline severity. The only statistically significant association between longitudinal severity and tract damage was observed for the uncinate fasciculus, but this did not survive correction for multiple comparisons (Table 4).

**Table 4:** Comparison of baseline and longitudinal aphasia severity between patients with above- and below-chance probability of tract disconnection. The two groups were defined using an atlas-based analysis on AP maps.

	**Above median (rank, n)**	**Below median (rank, n)**	**Test statistic**
***Arcuate fasciculus, long segment***			
*Aphasia severity (AQ), baseline*	10.14 (7)	6.12 (8)	U = 13, p = 0.94
*Aphasia severity (AQ), 6 months*	8 (5)	6.38 (8)	U = 15, p = 0.524
***Arcuate fasciculus, anterior segment***			
*Aphasia severity (AQ), baseline*	10.75 (8)	4.86 (7)	U = 6, p = 0.009
*Aphasia severity (AQ), 6 months*	8.83 (6)	5.43 (7)	U = 10, p = 0.138
***Arcuate fasciculus, posterior segment***			
*Aphasia severity (AQ), baseline*	9 (6)	5.29 (7)	U = 21, p = 0.463
*Aphasia severity (AQ), 6 months*	9 (7)	7.12 (8)	U = 9, p = 0.101
***Frontal aslant tract***			
*Aphasia severity (AQ), baseline*	14.5 (13)	7 (2)	U = 0, p = 0.019
*Aphasia severity (AQ), 6 months*	10.5 (11)	6.3 (2)	U = 4, p = 0.231
***Inferior fronto-occipital fasciculus***			
*Aphasia severity (AQ), baseline*	11 (12)	7.25 (3)	U = 9, p = 0.233
*Aphasia severity (AQ), 6 months*	6.3 (10)	9.3 (3)	U = 8, p = 0.287
***Uncinate fasciculus***			
*Aphasia severity (AQ), baseline*	10 (8)	5.71 (7)	U = 12, p = 0.72
*Aphasia severity (AQ), 6 months*	8.75 (8)	4.2 (5)	U = 6, p = 0.045

We observed a statistically significant correlation between baseline severity and likelihood of tract damage for the anterior (r = –0.70, p < 0.001) and long segment (r = –0.53, p = 0.042) of the arcuate fasciculus, and the frontal aslant tract (r = –0.57, p = 0.027). Only the correlation between severity at follow-up and likelihood of damage to the uncinate fasciculus (r = –0.6, p = 0.030) was statistically significant. However, only the correlation with the anterior segment survived correction for multiple comparisons.

**Fig. 5:**
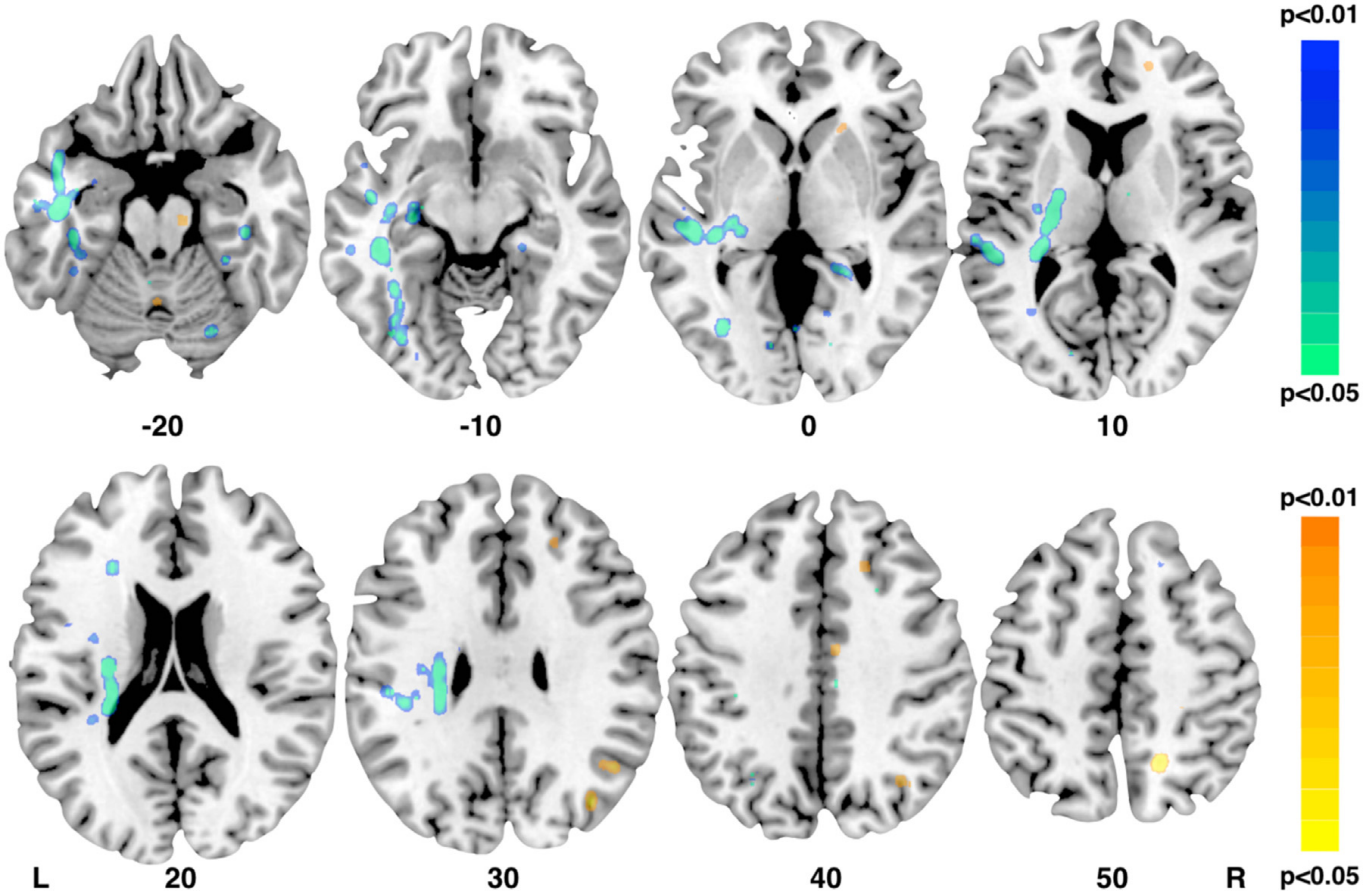
Tract-based spatial statistics (TBSS) analysis of acute imaging fractional anisotropy (FA) values and longitudinal language recovery (follow-up AQ). Cold colours indicate FA voxels negatively associated with longitudinal symptom severity; warmer colours indicate FA voxels positively associated with longitudinal symptom severity. Results are not corrected for multiple comparisons. Contrasts are controlled for age, sex, thrombolysis, and lesion size.

*TBSS* analysis indicated that for the left hemisphere reduced FA in the white matter of the temporal stem, anterior temporal lobe, fusiform gyrus, fronto-parietal white matter and posterior limb of the internal capsule was associated with more severe longitudinal symptoms. A smaller cluster was also identified in the posterior frontal lobe whereas no clusters within the anterior external capsule were significant. In the right hemisphere, smaller clusters of increased FA in the white matter of the superior frontal gyrus, the anterior limb of the internal capsule, the cerebral peduncle, the cingulate gyrus, and both inferior and superior parietal lobules predicted higher AQ scores at follow-up (Fig. 5).

The *tractography* analysis showed no statistically significant correlations between FA and volume of all tracts and aphasia severity at baseline for the left hemisphere. In the right hemisphere, correlations between baseline severity and FA of the frontal aslant tract and inferior fronto-occipital fasciculus were statistically significant but only the former survived correction for multiple comparisons. Correlation between longitudinal severity and tract volume of the right long segment of the arcuate fasciculus were statistically significant but did not survive correction for multiple comparisons. No other correlations were significant (Table 5).

**Table 5:** Tractography analysis of tract-specific measurements (volume and fractional anisotropy) in the left and right hemisphere and their association to symptom severity at baseline and chronic stage.

	**Left hemisphere**	**Right hemisphere**
**Aphasia severity at baseline**		
***Tract volume***		
*Arcuate Fasciculus, long segment*	r = 0.355, p = 0.162	r = 0.265, p = 0.305
*Arcuate Fasciculus, anterior segment*	r = 0.241, p = 0.465	r = −0.064, p = 0.807
*Arcuate Fasciculus, posterior segment*	r = 0.256, p = 0.321	r = −0.178, p = 0.493
*Frontal aslant tract*	r = −0.333, p = 0.192	r = −0.260, p = 0.314
*Inferior fronto-occipital fasciculus*	r = 0.120, p = 0.646	r = −0.016, p = 0.951
*Uncinate fasciculus*	r = 0.336, p = 0.188	r = 0.154, p = 0.554
***Fractional anisotropy***		
*Arcuate Fasciculus, long segment*	r = 0.168, p = 0.520	r = −0.028, p = 0.921
*Arcuate Fasciculus, anterior segment*	r = 0.100, p = 0.711	r = −0.190, p = 0.465
*Arcuate Fasciculus, posterior segment*	r = 0.033, p = 0.899	r = −0.001, p = 0.997
*Frontal aslant tract*	r = −0.115, p = 0.660	r = −0.637, p = 0.006
*Inferior fronto-occipital fasciculus*	r = 0.449, p = 0.071	r = −0.520, p = 0.033
*Uncinate fasciculus*	r = 0.117, p = 0.654	r = −0.151, p = 0.563
**Aphasia severity at six months**		
***Tract volume***		
*Arcuate Fasciculus, anterior segment*	r = −0.024, p = 0.933	r = 0.216, p = 0.438
*Arcuate Fasciculus, posterior segment*	r = 0.430, p = 0.110	r = 0.047, p = 0.869
*Arcuate Fasciculus, long segment*	r = 0.309, p = 0.260	r = 0.559, p = 0.03
*Frontal aslant tract*	r = 0.023, p = 0.934	r = −0.088, p = 0.756
*Inferior fronto-occipital fasciculus*	r = 0.228, p = 0.414	r = 0.180, p = 0.520
*Uncinate fasciculus*	r = −0.153, p = 0.586	r = 0.169, p = 0.547
***Fractional anisotropy***		
*Arcuate Fasciculus, anterior segment*	r = −0.485, p = 0.079	r = −0.047, p = 0.867
*Arcuate Fasciculus, posterior segment*	r = 0.043, p = 0.880	r = 0.055, p = 0.846
*Arcuate Fasciculus, long segment*	r = −0.063, p = 0.824	r = 0.046, p = 0.882
*Frontal aslant tract*	r = −0.195, p = 0.486	r = −0.348, p = 0.204
*Inferior fronto-occipital fasciculus*	r = 0.349, p = 0.202	r = −0.009, p = 0.974
*Uncinate fasciculus*	r = −0.251, p = 0.367	r = −0.069, p = 0.807

In a previous study we were able to demonstrate the value of studying the white matter in the contralesional hemisphere (Forkel et al., 2014). When only studying classical predictors of recovery, such as age, sex and lesion size, about 30% of the variance of recovery could be explained. However, when the volume of the right arcuate fasciculus was added to the model nearly 60% of the variance in recovery at six months could be explained.

**Fig. 6:**
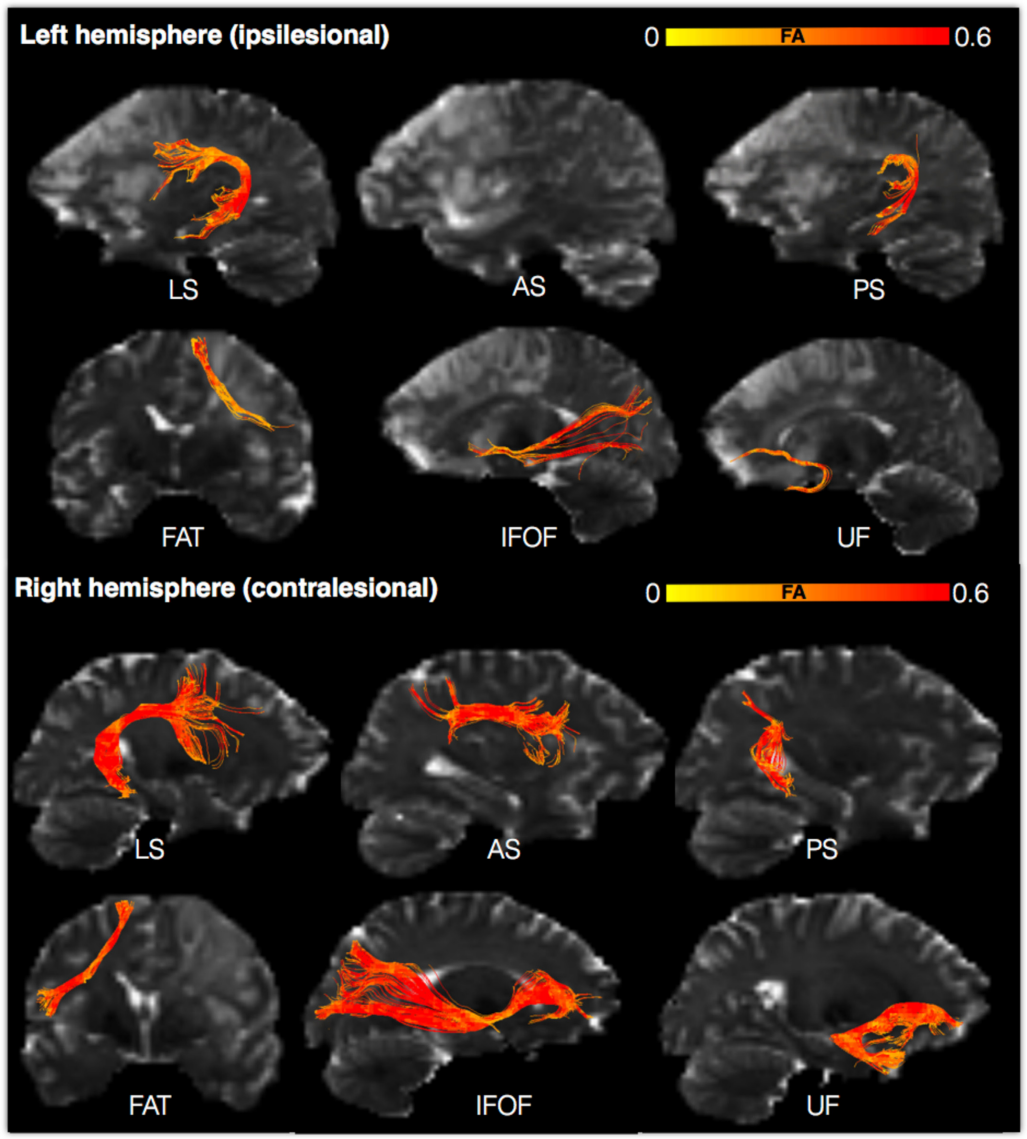
Left (ipsilesional) and right (contralesional) hemisphere white matter tractography reconstruction from a single aphasic stroke patient (male, 28 years, AQ baseline = 45, AQ follow-up = 96.2). The upper panel shows the lesioned hemisphere and the reconstructions of the language pathways within this hemisphere. The lower panel shows the same tracts in the non-lesioned hemisphere (right). The scalar index of fractional anisotropy (FA) is mapped along each pathway. Brighter colours indicate reduced FA. The tract volume in the left hemisphere was reduced for most of the pathways and the anterior segment was completely damaged. In the right hemisphere, all pathways were reconstructed, including the long segment of the arcuate fasciculus. LS, long segment of the arcuate, AS, anterior segment of the arcuate, PS, posterior segment of the arcuate, FAT, frontal aslant tract, IFOF, inferior fronto-occipital fasciculus, UF, uncinated fasciculus.

For the current study, we repeated the analysis for the other white matter pathways to assess if the predictive value of the contralesional tract volume is specific for the long segment of the arcuate fasciculus or extends to other tracts. The hierarchical regression analysis showed that a model including age, sex and lesion size was not predictive of longitudinal aphasia severity [R^2^ = 0.275, F(3,12) = 1.52, P = 0.260]. When the right hemisphere white matter tracts were added to the model, the predictive value of the model did not improve with the frontal aslant tract [R^2^ = 0.015, F(4,11) = 1.128, P = 0.393; R^2^ change: F(1,11) = 0.240, P = 0.634], the inferior fronto-occipital fasciculus [R^2^ = 0.065, F(4,11) = 1.259, P = 0.343; R^2^ change: F(1,11) = 0.039, P = 0.448], and uncinate fasciculus [R^2^ = 0.278, F(4,11) = 1.060, P = 0.421; R^2^ change: F(1,11) = 0.042, P = 0.842] Fig. 6.

## 4 Discussion

In this study, structural and diffusion imaging analysis of a longitudinal stroke dataset was carried out to i) map the extent and severity of white matter lesions and ii) identify anatomical predictors of language recovery using both voxel- and tract-based approaches. Mapping white matter lesions with a voxel-based approach identified a broader lesion overlap when diffusion data were used compared to structural images. The lesion identified by diffusion data extended to voxels that were significantly correlated with symptom severity at follow-up. The voxel-based analysis in combination with an atlas-based approach identified the tracts that were most likely affected by the lesions, nevertheless this analysis was underpowered to associate damage to individual tracts with longitudinal outcomes. Tractography analysis of diffusion data identified damage to individual tracts but was unable to detect statistically significant associations between tract-specific measurements (i.e. volume or FA) and aphasia recovery. The advantage of using a tractography-based approach was represented by the possibility of identifying additional mechanisms of recovery that involved contralesional tracts, in particular the long segment of the arcuate fasciculus.

While previous studies have compared different imaging approaches and modalities in stroke patients, this is the first study to assess on the same dataset voxel-based, atlas-based, and tractography methods that are commonly used to map white matter lesions in stroke. Our lesion overlay analysis showed that both structural and diffusion data converged in displaying a core region of maximum overlap but its peripheral extent varied according to the images used. In particular, diffusion analysis revealed significant differences in more posterior temporal and parietal white matter regions. These differences might be partially due to the low sensitivity of structural data to acute ischemic changes, especially for images acquired within the first 24 h (Birenbaum and Bancroft, 2011). This advantage of diffusion maps should not be generalised as the diffusion signal tends to vary over time in the subacute stage (Bykowski et al., 2004, Schlaug et al., 1997, Warach et al., 1995). Within minutes of an ischemic occlusion, lesioned tissue shows changes in diffusion signal intensity (Moseley et al., 1990, Pierpaoli et al., 1996), which remains abnormal for some time before the signal returns to a normal appearing level. This process of pseudonormalisation is due to the tissue regaining diffusion values within the normal range despite being nonviable or permanently damaged (Schlaug et al., 1997). Pseudonormalisation may last several days before the diffusion signal changes again to the pathological levels observed at later stages of stroke (Warach et al., 1995). While pseudonormalisation is not relevant in chronic stroke, it might pose a problem for acute imaging studies when patients are scanned at variable time points, which could result in a different estimation of the lesion volume depending on the time the scan was obtained.

While the considerations made above indicate that several mechanisms may explain differences between structural and diffusion results, the longitudinal analysis showed that the altered posterior white matter regions revealed by diffusion datasets are clinically relevant as they are associated to severity of longitudinal symptoms. Voxels that are detected as normal in the structural images but affected in diffusion data are more likely to contain salvageable white matter tissue and therefore their identification could have important treatment indications. The predictive value of the diffusion data is, however, highly dependent on the type of diffusion data and analysis. In our study, for example, the correlation between longitudinal symptom severity and frontal lobe white matter damage was more evident in the VLSM analysis than the TBSS. While in our study VLSM was performed using AP maps and TBSS was performed using FA maps, which might explain some of the differences observed between the results, other possible factors should be considered. A fundamental difference between the two methods is that VLSM is constrained to all voxels within the lesion masks whereas TBSS includes all brain voxels within a white matter skeleton that extend beyond the lesion (Smith et al., 2004, Smith et al., 2007). VLSM therefore requires a precise anatomical delineation of the lesions and is blind to changes occurring outside the lesion mask. This explains why VLSM results are driven primarily by areas of high overlay where statistical power is higher, and this is particularly true in small patient samples (Bates et al., 2003, Kimberg et al., 2007, Medina et al., 2010, Rorden et al., 2007a, Rorden et al., 2007b, Rorden et al., 2009, Rudrauf et al., 2008, Mah et al., 2014). TBSS automatically extracts a white matter skeleton either from the patients’ images or a matched control group. The skeleton is then applied to the individual maps to extract diffusion measures. These steps may introduce biases in stroke analysis as the voxels containing damaged white matter may either fall outside the voxels of the skeleton or prevent extracting diffusion values from those parts of the skeleton severely affected by the lesion (Jones et al., 2010). Unfortunately, there are only a few studies that have used TBSS in stroke and those vary in their methodological approaches used to prevent these limitations (Geva et al., 2015; Lunven et al., 2015).

In our study, an atlas-based analysis was used to indirectly identify and quantify damage to specific tracts. Both structural and diffusion analysis revealed extensive white matter damage to most left hemisphere tracts, although the prevalence of tract damage was higher for the diffusion data. Correlations were significant only with baseline AQ measures but not with longitudinal aphasia severity. The lack of sensitivity to language recovery of the atlas-based approach may have different explanations, including misregistration, demographic and anatomical mismatch between normative data used to generate atlases and our patient population, and interindividual variability in tract anatomy. Indeed, in our study four patients were calculated to have a significant disconnection of the optic tract, yet not a single patient in our study was blind before or after their stroke. In addition, the small number of subjects in our study is a clear limitation of the atlas-based approach as a small number of subjects belongs to the group with ‘low or no disconnection’. There are other limitations of the atlas-based approach that are common to all datasets and, therefore, are important to consider when interpreting the results. The atlas-based approach relies on tract probability masks that in the majority of cases are generated from young healthy control participants that are not matched to the patient population (Catani and Thiebaut de Schotten, 2008, Hua et al., 2008, Rojkova et al., 2015, Wakana et al., 2004). Furthermore, atlases vary according to the tracking algorithms, automatic vs. manual delineation of regions of interest, sample size used to calculate maps, and the quality of the data used to generate the tracts. Finally, the atlas-based approach does not allow for directly relating tract damage to specific symptoms as they do not provide quantification of the severity of tract abnormalities. Indeed, what is commonly considered as a ‘probability of disconnection’ is only a probability of a co-localisation between the lesion mask and the tract maps. There are other atlas-based approaches that were not used in our study. For example, average percentage maps of individual tracts from healthy subjects can be used as masks to extract diffusion measurements or other measurements from the datasets of individual patients (Catani et al., 2013a, Catani et al., 2013b, Ivanova et al., 2016). This approach reduces the operator-dependent bias as it uses the same mask for all subjects. However, one inconvenience of this approach is the lack of proxy measures of volume atrophy, which can be obtained only with a tractography analysis of diffusion images acquired directly from patients.

Tractography is the only method that can visualise tract anatomy in individual patients and extract specific measures of tract volume and microstructural organisation. In our study, we dissected individual language pathways and compared left and right differences to identify reduced volume and FA in tracts of interest. This approach accounts for pre-stroke inter-individual differences in tract anatomy for bilateral and symmetrical pathways as it uses the same pathway in the contralesional hemisphere as control. Language pathways that have been demonstrated to have a bilateral volume in the adult brain include the IFOF, the UF, and the posterior segment of the arcuate (Forkel et al., 2014; Catani et al., 2007; López-Barroso et al., 2013; Thiebaut de Schotten et al., 2011). Other tracts, such as the anterior and long segments of the arcuate fasciculus, and the frontal aslant tracts, present a more asymmetrical pattern, which makes the results of their volume analysis difficult to interpret (Catani et al., 2007, Catani et al., 2012a, Catani et al., 2012b, Lopez-Barroso et al., 2013, Thiebaut de Schotten et al., 2011). However, for all language tracts but the anterior segment, FA is bilateral in the normal brain and therefore FA results of the left and right comparisons are more indicative of tract damage. Indeed, the FA analysis showed reduced values for language tracts in the left hemisphere except for the frontal aslant tract. An alternative approach commonly used in tractography studies is the use of control subjects matched for sex, age, handedness, and education (Catani et al., 2013a, Catani et al., 2013b, Geva et al., 2015), which we were not able to perform in this study. Despite the advantages of tractography, our longitudinal analysis failed to demonstrate an association between damage to specific tracts in the left hemisphere and aphasia severity at follow-up. Tracking in the presence of an acute stroke lesion can be difficult due to reduced FA, and may generate either false positive (i.e. aberrant streamlines) or false negative (i.e. not visualising streamlines when they are present) reconstructions (Dell'Acqua and Catani, 2012), which ultimately can reduce the sensitivity of tract-specific measurements.

The use of alternative methods of tracking (e.g. probabilistic tractography) can help to reduce the number of false negatives but the specificity of the diffusion measurements also reduces due to the higher number of false positives (Dell'Acqua and Catani, 2012). At the same time pseudonormalisation may overestimate the volume of damaged tracts in the acute phase and reduce the possibility of detecting direct correlations with longitudinal measures. Whilst FA is an index widely used in diffusion imaging, other measures, for example hindrance modulated orientational anisotropy (HMOA), is able to identify white matter changes that are not detectable with conventional indices (Dell’Acqua et al., 2013). Using such advanced models in stroke may also allow to separate damaged tissue in different classes by analysing data acquired with multiple diffusion-weighting at different b-value (e.g., multishell data).

Additionally, the aphasia quotient is a composite score that may not necessarily correlate with damage to a specific tract but rather to a network of multiple tracts. It was beyond the scope of this study to correlate anatomy of individual tracts to scores of subcomponent contributing to the calculation of the AQ but future analysis of larger datasets may reveal specific correlations between subtypes of aphasia and single tracts or multiple networks as shown in recent papers adopting connectomic approaches to aphasia patients (Fridriksson et al., 2018). It is also important to highlight that dynamic changes occur after stroke and the functional effects of the lesion may involve extended networks beyond the classical language networks (Geranmayeh et al., 2017).

Finally, both TBSS and tractography analysis indicates the importance of the right hemisphere white matter anatomy for language recovery. This is in line with the findings from previous functional imaging and clinical studies that have suggested the recruitment of right hemispheric homologues of language areas as a possible compensatory mechanism (Crinion and Price, 2005, Leff et al., 2002, Musso et al., 1999, Rosen et al., 2000, Saur et al., 2006, Sharp et al., 2004, Thiel et al., 2006, Weiller et al., 1995; Price et al., 2017). Early studies indicated a compensatory role of the right hemisphere in patients who recovered their language after a left-hemispheric stroke and later became aphasic again, following a right-hemispheric stroke (Nielsen, 1946) or as a consequence of temporary right-hemispheric anaesthesia (i.e. Wada test) (Cappa et al., 1997, Kinsbourne, 1971, Ohyama et al., 1996, Thulborn et al., 1999, Wada et al., 1975). Overall, these and other studies suggest that recovery of language after stroke is a dynamic process in which the right hemisphere is important for longitudinal outcomes. However, the right hemisphere involvement is heterogeneous as not all aphasic patients benefit from this mechanism. In a previous study, we demonstrated that only those patients with a larger long segment of the arcuate fasciculus in the right hemisphere are likely to recover language (Forkel et al., 2014).

In conclusion, recovery of language rely on a complex dynamic between ipsi- and contralesional mechanisms in which both anatomical and functional changes may play a significant role. Future studies combining conventional lesion mapping with functional and tractography approaches in larger cohorts of patients are necessary to understand the relationship between structural and functional mechanisms of recovery in both hemispheres.
